# Response Assessment to Erythropoietin-Zeta (Epo-Alpha Biosimilar) Therapy in Low-Risk Myelodysplastic Syndromes

**DOI:** 10.3390/jcm11061665

**Published:** 2022-03-17

**Authors:** Calogero Vetro, Valeria Di Giacomo, Donato Mannina, Silvana Magrin, Antonio Mulè, Maria Enza Mitra, Sergio Siragusa, Andrea Duminuco, Bruno Garibaldi, Maria Cristina Emanuela Vadalà, Francesco Di Raimondo, Giuseppe A. Palumbo

**Affiliations:** 1 Division of Hematology, A.O.U. Policlinico “G. Rodolico-San Marco”, 95123 Catania, Italy; c.vetro@policlinico.unict.it (C.V.); m.cristinavadala@gmail.com (M.C.E.V.); diraimon@unict.it (F.D.R.); 2 Department of Hematology, Azienda Ospedaliera Papardo, 98158 Messina, Italy; digiava@libero.it (V.D.G.); donamanni@gmail.com (D.M.); 3 BMT Unit, Division of Hematology, V. Cervello Hospital, 90146 Palermo, Italy; silvana.magrin@libero.it (S.M.); a.mule@villasofia.it (A.M.); memitra@yahoo.com (M.E.M.); 4 Hematology Unit, Thrombosis and Hemostasis Reference Regional Center, University of Palermo, 90127 Palermo, Italy; sergio.siragusa@unipa.it; 5 Postgraduate School of Hematology, University of Catania, 95123 Catania, Italy; andrea.duminuco@gmail.com (A.D.); brunga93@gmail.com (B.G.); 6 Department of Scienze Mediche, Chirurgiche e Tecnologie Avanzate “G.F. Ingrassia”, University of Catania, 95123 Catania, Italy

**Keywords:** myelodysplastic syndromes, anemia, erythropoietin, biosimilar pharmaceuticals

## Abstract

Background. This prospective observational study aimed to verify the efficacy of erythropoietin zeta in the treatment of patients with low-risk myelodysplastic syndrome. Methods. Patients with low/int-1 IPSS risk and serum erythropoietin level below 500 U/L were enrolled. Treatment consisted of erythropoietin zeta 40,000 U subcutaneously once a week. The primary endpoint was the erythroid response. According to Simon’s two-stage statistical design, 36 patients were recruited. The median age was 75 years (range 56–83 years), male/female ratio was 1.1/1, median baseline serum erythropoietin was 57.9 U/L (range 9.4–475 U/L). 53% of patients had low-risk disease, while the remaining had Int-1 risk. Results. After 8 weeks, a significant response (rise in Hb levels of at least 1.5 g/dL) was achieved in 18 patients (50%) out of 36. However, 17 patients did not improve; 8/17 patients pursued the 40,000 U weekly schedule of erythropoietin zeta, and 4/8 (50%) of them reached the erythroid response after 16 weeks. Nine patients underwent dosage doubling (40,000 U twice per week), and 5/9 (55%) of them achieved the erythroid response. Conclusion. Compared with data from the literature, this prospective study revealed that EPO-zeta is a safe and effective therapeutic option in low-risk MDS patients.

## 1. Introduction

Myelodysplastic syndromes (MDS) are a group of heterogeneous clinical conditions characterized by ineffective hematopoiesis, dysplastic changes, and one or more peripheral blood cytopenias (anemia, thrombocytopenia, neutropenia) [1].

Apart from neutropenia and thrombocytopenia, anemia is a prominent feature limiting the quality of life of these patients [2] and worsening the end-organ damages, in particular the cardiac performance [3]. To date, supportive care with erythropoietin-stimulating agents (ESA) has demonstrated higher efficacy compared to placebo [4,5], and is widely used for the treatment of low-risk MDS patients [6,7] (i.e., patients with IPSS score low/intermediate-1 (int-1)), leading to an erythroid response in up to 58% of patients [8,9]. The achievement of higher hemoglobin levels is of pivotal importance in the context of transfusion-dependent and transfusion-independent MDS-related anemia [10]. Recent studies in a real-life setting have highlighted that high hemoglobin levels improve the quality of life of affected patients [11] and cardiac performance [10]. In the pivotal study by Oliva et al. [3], the achievement of hemoglobin levels higher than 10.7 g/dL resulted in a positive effect on cardiac performance in low-risk MDS patients; in particular, the cardiac remodeling was lower in the group of patients with levels of Hb above the threshold mentioned above. Moreover, guaranteeing transfusion independence and constantly high levels of Hb leads to a rationale in the pathophysiology of anemia-related heart disease [12]. Indeed, transfusion is not usually allowed in cases with Hb greater or equal to 7.5–8.5 g/dL, except for acute symptoms [13]. Thus, even if transfusion is effective, patients would experience an intermittent Hb augmentation [14,15]. Additionally, the transfusion-related iron overload could worsen the end-organ damage, especially in the liver and heart [16]. As a result, transfusion independence determines a more prolonged survival for patients [17].

Recently, biosimilar ESAs have entered the clinical practice management of these patients [18]. However, few studies are related to the effectiveness of biosimilar ESAs in the specific treatment of low-risk MDS. Data are extrapolated from the presumed equal efficacy with the originator in renal insufficiency-related anemia [19] and chemotherapy-induced anemia [20]. 

To assess the effectiveness of these agents, we started a prospective study to evaluate the response rate to a biosimilar ESA, namely EPO-zeta, and its safety.

## 2. Design and Method

### 2.1. Study Design

This was a prospective, observational, not-for-profit, and multicenter study. Four different Haematological Centers located in Sicily, Italy, participated in the study. The study was approved by the local ethics committees and conducted according to the relevant national and local guidelines in the Helsinki declaration. Enrollment started in October 2012 and ended in March 2015.

The study aimed to assess the response rate to EPO-zeta (Retacrit^®^, Hospira, Lake Forest, IL, USA), a biosimilar of EPO-alpha, in low-/intermediate 1-risk MDS patients who are eligible for supportive care with erythropoietin.

The primary endpoint was the response rate to biosimilar ESA. The secondary endpoint was to estimate drug safety. 

We applied Simon’s two-stage design for sample size calculation. This design allows one to stop the study if the minimum required percentage of responses is not met in a pre-defined fraction of the total number of patients to be enrolled. Otherwise, enrolment continues until the total number of planned patients is reached. 

### 2.2. Patients

Patients with low/int-1 risk MDS defined by IPSS [21] were eligible for the study. The diagnosis was based on the 2008 WHO classification of a tumor of hematopoietic and lymphoid tissues, and was confirmed within 6 months before treatment started. Previous therapy with ESAs was not allowed. Values of hemoglobin lower than 10 g/dL were required for treatment to start. Baseline serum erythropoietin (sEPO) dosage was required for each patient, and those with sEPO levels greater than 500 U/L were excluded [22]. Exclusion criteria were also a performance status greater than 2, according to ECOG, uncontrolled cardiovascular diseases (particularly hypertension) and anemia due to chronic inflammatory disease, or chronic hemorrhage or hemolysis. 

MDS patients with intermediate-2 or high-risk MDS according to IPSS, patients with secondary or therapy-related MDS, bone marrow fibrosis (defined as MF-2), and patients previously treated with ESA, serum EPO levels > 500 MU/mL, with a high transfusion requirement (4 or more units of PRBC within 8 weeks before the start of EPO-zeta) before the administration of EPO-zeta, were not enrolled.

### 2.3. Treatment Flow

Bone marrow aspirate and/or bone marrow biopsy and cytogenetic examination, blood count, liver and renal function tests, iron status (serum iron, transferrin, and ferritin), LDH, vitamin B12, and folate dosage, and dosage levels of serum erythropoietin were mandatory before starting the treatment. 

Possible deficiencies of iron, vit. B12 and/or folate were corrected before the initiation of EPO-zeta administration [6,23]. 

After performing a baseline assessment and signing informed consent, therapy with EPO-zeta was started at a dose of 40,000 U/week by subcutaneous administration [24]. The response was assessed after 8 weeks. If the patient showed an erythroid response, according to IWG 2006 criteria [25], the treatment was pursued. If no response was achieved, a doubling dosage with 40,000 U/day for 2 consecutive days/week (i.e., 80,000 U/week) 20 was permitted. However, as a general rule, if patients had a trend toward Hb augmentation (i.e., Hb increase of at least 0.5 g/dL), they kept on with weekly 40,000 U, while if the increase was less than 0.5 g/dL or absent, the dosage was doubled. The drug was interrupted if Hb levels were greater than 12 g/dL, and then resumed when Hb was below 11 g/dL. Treatment response was assessed at 8, 16, and 24 weeks from the start of treatment. 

### 2.4. Response Evaluation

Erythroid response by the 2006 International Working Group (IWG) criteria [25] in patients with pretreatment Hb below 11 g/dL is present when either there is a decrease of at least 4 units of transfused PRBC in eight weeks compared to those transfused in the same time with pre-treatment values of Hb < 9 g/dL (not applicable to our data set), and/or an increase in Hb of at least 1.5 g/dL, re-checked in two subsequent samplings and maintained over time. Harmful and undesired effects resulting from the medication were classified according to the CTC-AE v.4.

### 2.5. Statistical Analysis

Given that the rate of erythroid response to treatment with the originator is reported to be 50–58% [8,26], the hypothesis for the current study was that the biosimilar drug was also effective in determining a response rate of at least 55%. 

For descriptive statistics, data are reported as median and range or average and standard deviation. Dichotomous variables were compared using χ^2^-test and continuous variables using Student’s *t*-test or Mann–Whitney Test in case of parametric and non-parametric statistics, respectively. The Kaplan–Meier method was applied for survival curves, and results were analyzed using the two-sided log-rank test. Overall survival was calculated from diagnosis to last follow-up or death. 

All statistical tests were two-sided, and statistical significance was set at *p* < 0.05. Statistical analyses were performed using Graph Pad Prism version 5.00 for Windows (Graph Pad Software, San Diego, CA, USA) or SPSS version 20 (IBM Corporation, Armonk, NY, USA).

### 2.6. Primary Endpoint 

To verify the efficacy of EPO-zeta in the treatment of patients affected by myelodysplastic syndrome with low/int-1 risk according to IPSS score, we assessed the rate of subjects reaching the erythroid response according to the criteria of the IWG published in 2006 (an increase of Hb of at least 1.5 g/dL) during a treatment period of 24 weeks [25].

### 2.7. Sample Size

The null hypothesis (an ineffective drug) is erythroid response rate ≤55% (p0); the alternative hypothesis (drug effective) is erythroid response rate ≥75% (p1). According to Simon’s two-stage statistical design (minimax variant), the drug is effective if the response is shown in 15 out of the first 24 enrolled patients. Finally, the response should be documented in 24 out of a total of 36 evaluable patients. 

Data have been set to obtain an 80% probability (1-β) of accepting the alternative hypothesis (type II error) and 95% probability (1-α) (type I error) of receiving the null hypothesis if the latter is indeed true. The likelihood of early termination was 0.827.

### 2.8. Secondary Endpoint

Post-hoc analysis to evaluate the safety Hb threshold achievement rate at the end of the study. At week 24, patients were assessed to verify if they would have reached levels of Hb greater than 10.7 g/dL. Such a threshold has been based on literature data and is known to reduce the rate of cardiac remodeling [3].

To verify EPO-zeta safety, collect any adverse events (AE) reported during the treatment and compare them to those reported in the literature for similar patients during the treatment period (24 weeks). AEs were scored according to the National Cancer Institute Common Terminology Criteria for Adverse Events (version 4.0). 

## 3. Results

### 3.1. Patient Characteristics

All 36 evaluable patients (19 males and 17 females) underwent treatment with EPO-zeta. The median age at enrollment was 75 years (range 56–83 years). Median hemoglobin at baseline was 9.6 g/dL (range 7.8–10 g/dL); median serum erythropoietin was 57.9 U/L (range 9.4–475 U/L). A classification of MDS was performed according to the 2008 WHO classification [1]. IPSS risk stratification was performed according to [21], with 19 patients at low risk, and 17 at int-1 risk. All patients had a normal karyotype, except two harboring trisomy 8 as a sole cytogenetic abnormality. In two cases, ringed sideroblasts were identified. Table 1 indicates the disease and risk evaluation for each patient. All patients were followed for 24 weeks to evaluate the response to the therapy and the drug’s safety. For each patient, sero-virological and cardiac assessments were performed at baseline. CIRS was calculated for each patient. The median severity index was 0.3 (range 0.15–3.5), with 3 patients having 1 severe comorbidity and 2 patients having 2 and 3 severe comorbidities.

### 3.2. Erythroid Response

According to Simon’s two-stage design, an interim analysis was performed on the first 24 patients enrolled. These patients were enrolled from October 2013 to March 2014. Twelve patients showed an erythroid response after 8 weeks of treatment. Of the remaining 12 patients, 7 underwent a doubling in dosage because the Hb levels augmentation was inferior to 0.5 g/dL, and 2 of them eventually showed an erythroid response at week 16. Five patients pursued the weekly schedule, and 3 of them showed an erythroid response at week 16. Thus, 15 patients showed an erythroid response among the first set of 24 enrolled patients, and therefore, we pursued the study accrual, reaching a total of 36 patients. 

**Figure 1:**
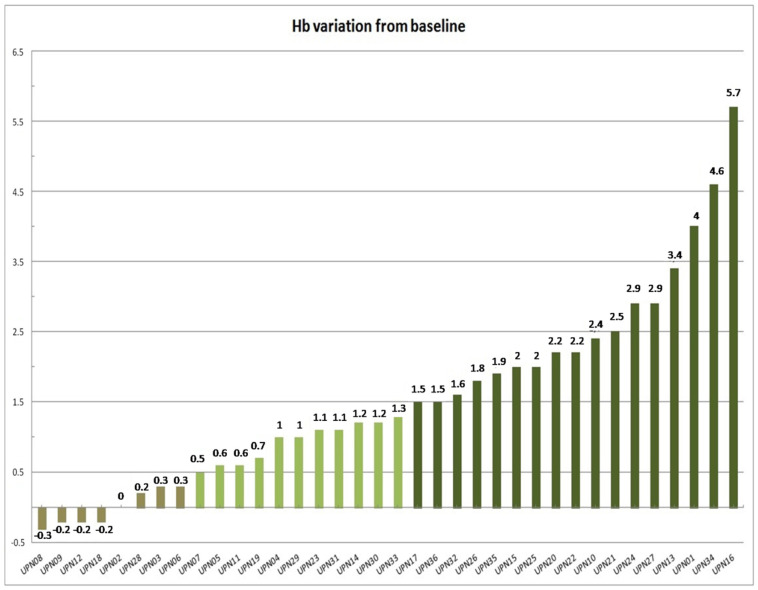
Waterfall plot indicating the Hb variation at 8th week from treatment start. Dark green indicates patients responding (Hb greater than 1.5 g/dL) at this time. Light green indicates patients who showed a Hb increase of at least 0.5 g/dL (but less than 1.5 g/dL). Brownish green indicates patients who showed Hb reduction or less than a 0.5 g/dL raise.

Considering the entire cohort, a erythroid response was achieved in 18 patients (50%) at week 8 (Figure 1). In the absence of any transfusional need, all responses were given by Hb rise of at least 1.5 g/dL. Refractoriness was documented in 18 patients (50%). Nevertheless, the Hb trend showed an upward trend in all except 4 patients (11.1%), where no Hb increase was reported.

**Figure 2:**
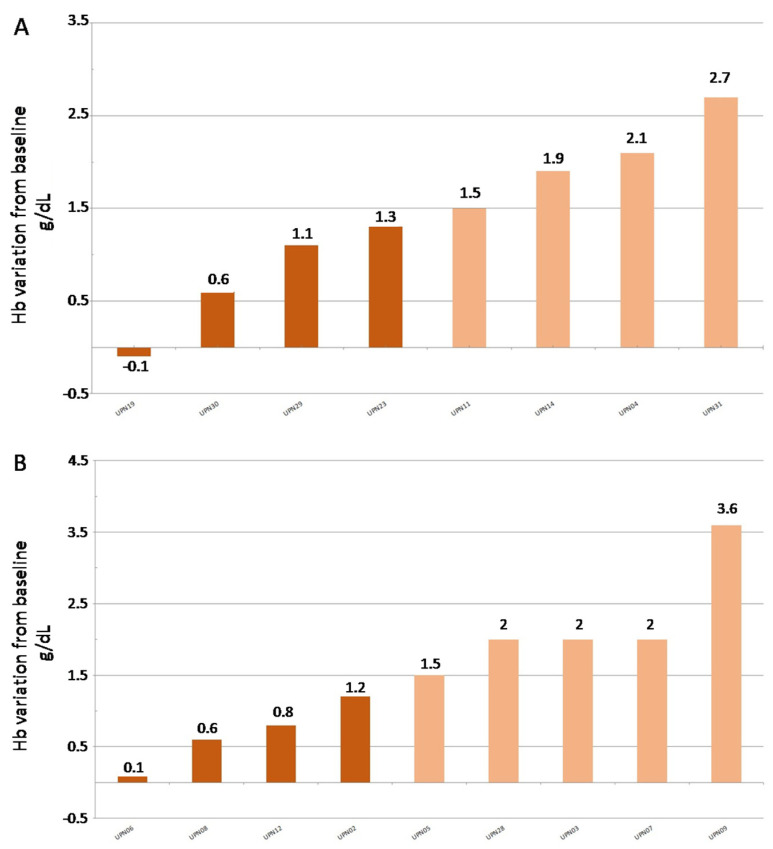
Waterfall plot indicating the Hb augmentation. (**A**) patients pursuing with 40,000 U of EPO-zeta per week; (**B**) patients doubling the dosage of EPO-zeta to 80,000 U per week.

At week 16, 17 refractory patients were evaluable (1 patient was lost-to-follow-up). Eight patients proceeded with the same dosage (Figure 2A), and four patients (50%) responded, while four patients (50%) remained refractory to the ESA. Nine patients (53%) underwent ESA doubling (Figure 2B), and five of them (55%) responded. The remaining four (45%) were still refractory to the treatment. 

**Figure 3:**
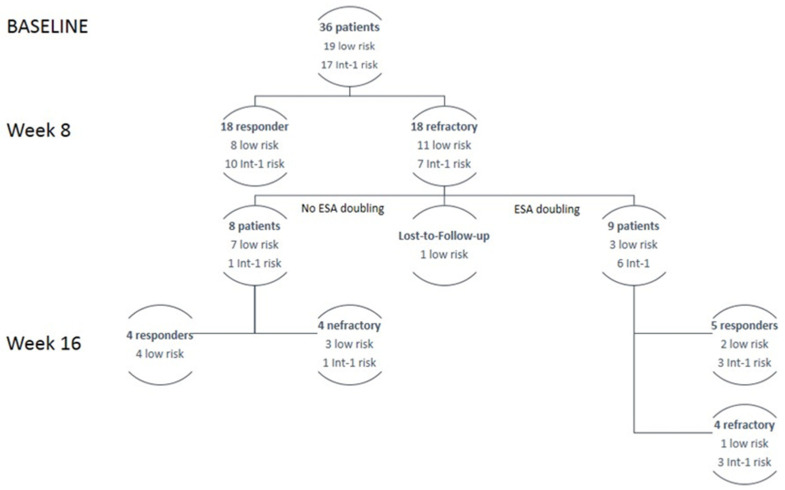
Patients flow according to treatment response and risk factors. ESA: erythropoietin stimulating agent.

Overall, 27 responses (75%) were evaluable after 16 weeks of evaluation. Figure 3 shows the response to the ESA according to disease characteristics. According to the study design, the study’s primary endpoint was reached. 

**Figure 4:**
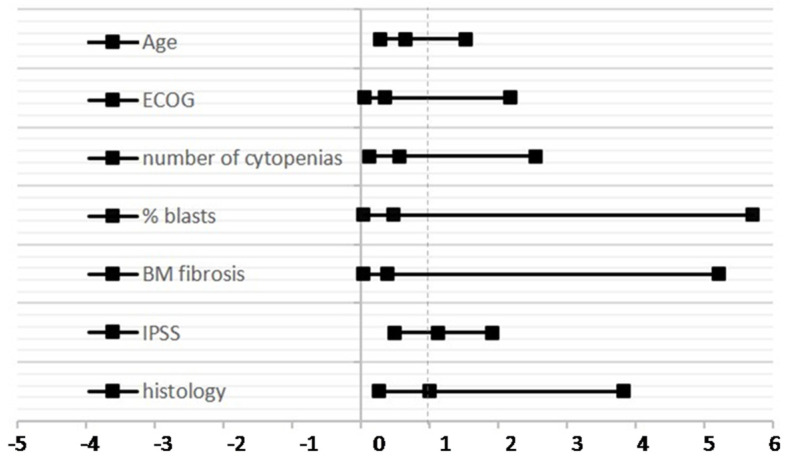
Forest plot indicating the relative risk according to clinical factors and response to ESA.

Going deeper into the response, we analyzed the impact of age (70 years vs. >70 years) at treatment start, ECOG performance status (0–1 vs. 2), percentage of BM blasts (<5% vs. 5–10%), number of cytopenias (0–1 vs. 2–3), IPSS (low vs. int-1), and histology (fibrosis vs. no fibrosis) (Table 2, Figure 4). However, we could not identify any clinical factors related to refractoriness.

### 3.3. Erythroid Response at Week 24

At 24 weeks, the Hb target of 10.7 was reached on average for all evaluable patients (30 out of 36, 83%). Indeed, evaluable patients showed overall a mean Hb of 10.7 ± 1.3 g/dL. In particular, patients responding at week 8 had a median of 10.8 ± 1.6 (group A), with 8 patients out of 13 (61%) with Hb greater than 10.7 g/dL, while those who underwent dosage doubling had a median of 11.1 ± 2.5 (group B), with 4 patients out of 9 (44%) with Hb greater than 10.7 g/dL; patients who did not undergo doubling had a median of 10.0 ± 1.5 (group C), with only 2 patients out of 8 (25%) having Hb greater than 10.7 g/dL.

**Figure 5:**
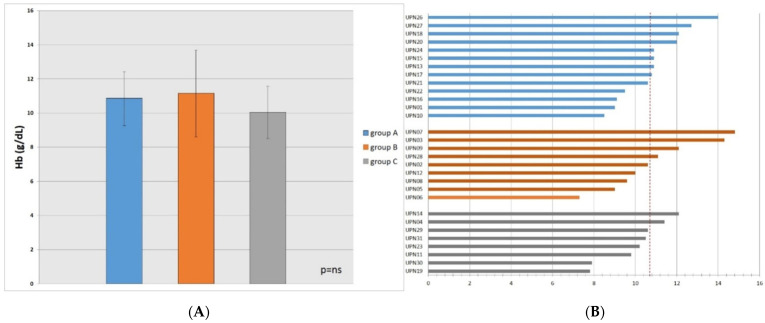
(**A**) Hb levels at week 24th in: group A (patients responding at week 8th), group B (patients who underwent dosage doubling), group C (patients who did not undergo doubling); (**B**) Hb levels at week 24 for each patient. A dashed line indicates a cut-off of Hb level at 10.7 g/dL. ns: not significant.

However, differences in Hb were not statistically significant (Figure 5A). Therefore, pursuing the 40,000 U dosage in patients showing a Hb increase greater than 0.5 g/dL could be considered a valid option (Figure 5B). Similarly, doubling dosage for very refractory patients is a recommendable strategy. Therefore, erythropoietin zeta is a valuable tool to reach an Hb target of 10.7 g/dL, which can be considered a reasonable threshold. 

### 3.4. Safety 

The safety profile was analyzed. Adverse events, possibly related to the study drug, are listed in Table 3. Overall, 9 AEs were registered. Four occurred during the weekly schedule, while 5 occurred after doubling the drug dosage. Grade 1/2 was observed in 12% of cases. In one case, the development of glucose intolerance requiring appropriate treatment was reported; mild hypertension increased in one patient, transaminases augmentation (less than 3 UNL) in another patient, and in one case, a headache, sensitive to analgesics, was observed. The most frequent grades in 3/4 AEs were infections (2 patients, 5.5% of study population) and hypertension (2 patients; 5.5% of the study population, requiring more than one anti-hypertensive drug). It should be considered that the two patients that developed grade 3/4 hypertension suffered from this disease before treatment initiation. In particular, one of them suffered from chronic heart failure and underlying type II diabetes mellitus. The other patient had an aortic dilatation and pulmonary hypertension. In both cases, grade 3 hypertension arose after doubling EPO administration because of absent response to 40,000 U/week. Moreover, it should be highlighted that three other enrolled patients had essential hypertension before study initiation, and in none of them, this condition worsened during the entire study period. 

### 3.5. Long Term Follow-Up

Even if not included in the study’s primary endpoint, we evaluated the long-term follow-up of 24 patients out of the 36 enrolled. The remaining 12 patients were lost to follow-up.

After a median follow-up of 5.3 years, the 5-year overall survival (OS) rate was 89%. One patient that responded to erythropoietin treatment 40,000 U/week, out of 24 (4%), progressed to acute myeloid leukemia, seven years after MDS diagnosis. One patient, who did not respond even to EPO doubling, developed alloimmunization, treated with immunosuppression. One patient that reacted only to EPO doubling died cause of a cardiac event, 6.8 years after MDS diagnosis. 

Furthermore, 18 out of these 24 patients (75%) responded to EPO-z at w16. Only 2 out of these 18 patients (11%) had a lost response to EPO-z, one after one year, and the other after seven years.

## 4. Discussion

In the literature, several studies have been focusing on the response to ESAs in low-risk MDS. The first reports indicated a low response rate (up to 38%) using EPO alpha at 40,000 U/weekly [27,28]. Ross et al. [29] summarized these studies, and found that 38% of patients will obtain a significant response; however, enrollment criteria and the variability in response definition could have played a role in these not enthusiastic results. After that, response assessment criteria were defined again in 2006 [25]. The first meta-analysis by Moyo et al. [8] reported a response rate up to 57.6%. Similarly, Mundle et al. found a response rate of 50% [26]. Furthermore, EPO-alpha and darbepoetin seem not to differ in inducing an erythroid response. In addition, ESA usage has been demonstrated to be safe [6]. 

Since the originator EPO-alpha (Eprex^®^, Jannsen, Beerse, Belgium) patent expired in 2007, biosimilars were proposed for the clinical management of anemia [30]. Because there is only one known receptor of erythropoietin, the drug’s mechanism of action is strictly related to its composition (European Medicines Agency. Guidance on similar medicinal products containing recombinant erythropoietin, 2006. Available from: https://www.ema.europa.eu/en/documents/scientific-guideline/guideline-non-clinical-clinical-development-similar-biological-medicinal-products-containing_en-1.pdf, accessed on 19 January 2022). Indeed, no cross-reactivities were expected. However, the source of variations compared to the originators are several, and, probably, it would not be correct to automatically ascertain that the biosimilar is as effective as the originator [31]. Indeed, some biological differences can exist, even related to the manufacturing processes. However, these differences are minimal, and do not affect the erythroid response in in vivo models [30]. Originator EPO-alpha formulation has 12% more monomers and more EPO protein than EPO-zeta (even if it did not reach statistical significance). At the same time, there are no differences regarding the molecular weight and isoforms of EPO itself. More interestingly, the in vivo potency of the biosimilar was not inferior to that of the originator [24,30]. 

In oncology, the first authorized biosimilar (HX575) showed an effective and safe profile superimposable with the originator [20]. Simultaneously, several biosimilars have been introduced in hematology [18], where their usage is justified by the principle of data extrapolation [31]. In other words, since the drug has been demonstrated to be effective in renal anemia, its usage could also be extended to other indications [31]. 

For these reasons, we decided to conduct a prospective observational study to verify the effectiveness and safety of the drug in MDS patients. Our results demonstrate that the biosimilar EPO- zeta, given weekly at 40,000 U s.c., effectively induces an erythroid response (based on the 2006 IWG criteria). Indeed, 50% of patients showed a response to the biosimilar at 8 weeks, while some of the remaining patients responded while keeping on with the treatment, either at the starting dose, or doubling it (40,000 U twice per week). These data overlap with those of other series. In the study by Park et al., evaluating 403 low-risk MDS patients, the erythroid response was evident in 50% of patients treated with EPO alpha or beta or darbepoetin [32]. In our patients set, we decided to double the EPO-zeta dosage when the Hb values would not have increased by at least 0.5 g/dL after 8 weeks of treatment. Considering all the patients enrolled in the study, 50% of patients achieved the erythroid response. It was already reported by Terpos et al. that a response to erythropoietin could be reached later during the treatment period, without increasing the drug dosage [33]. However, it should be said that this study was conducted before the publication of the IWG criteria, so a strict comparison cannot be made. Recently, the FISM group published a retrospective propensity-matched cohort study comparing patients treated with standard-dose erythropoietin (i.e., 40,000 U/week) and high-dose erythropoietin (i.e., 40,000 U twice weekly), finding that in general, there are no differences in erythroid response rate between these two groups, except in transfusion dependence and patients with worse prognostic features, both of these not the target of our study [34]. However, the patients who applied the dose doubling had no or minimal increases in Hb levels when treated with a standard erythropoietin dose. In response to and need for a higher amount of erythropoietin, this patient subset could mimic the patients with the most increased transfusional need. Finding markers predictive of possible response to higher doses of erythropoietin is, to date, an unmet clinical need, requiring more detailed studies. 

Moreover, it should be considered that after 24 weeks, most patients reached an Hb level of at least 10.7 g/dL. This is a crucial threshold, since cardiac performance is significantly affected by Hb levels, especially in elderly patients. The state of chronic anemia leads to cardiac remodeling, with consecutive cardiac hypertrophy [3]. Moreover, Hb increase translates to the amelioration of the quality of life and the increased overall survival of these patients [2,35,36,37]. In our opinion, the fact that, after 24 weeks, 83% of our patients can reach this safety threshold is additional proof of the effectiveness of erythropoietin zeta in the treatment of low/int-1-risk MDS patients, in line with data from real-life experiences [38,39]. 

As a final consideration, the drug is safe. Only nine AEs were registered in the patient set, with a total of 5 grade 3/4 adverse events (14% of the study population). The most frequent AE was hypertension, manageable with oral treatment. However, we saw this complication only in patients already suffering from cardiovascular diseases. Therefore, a direct link with ESA treatment cannot be drawn. This is also supported by the literature since, until now, no reports have indicated a linkage between ESA usage and hypertension [6]. Otherwise, the incidence of bacterial infection is probably due to the presence of MDS itself [40].

Thus, biosimilar erythropoietin-zeta is a valid and safe alternative to branded erythropoietin. Its usage would guarantee clinical results and patient quality of life, while sparing health care system resources—biosimilars are usually cheaper than their originators. However, we are aware that more extensive series and follow-ups are needed to better define the effectiveness and safety in the long run. 

## 5. Conclusions

In this prospective observational study, our group demonstrated that biosimilar erythropoietin effectively induces an erythroid response in low-risk MDS patients while maintaining a good safety profile, similar to that of the originator molecule. 

## Figures and Tables

**Table 1:** Characteristics of the disease according to the patient enrolled and risk stratification.

Patient ID	Gender	MDS Subtype	BM Blasts	BM Fibrosis	Perypheral Cytopenias	Karyotype	Full Karyotype	IPSS Score	Risk Category
1	M	RCMD	<5%	NOT EVALUATED	0/1	GOOD	46, XY [20]	0	Low
2	F	RCMD	<5%	ABSENT	2/3	GOOD	46, XX [20]	0.5	Int-1
3	F	RCMD	<5%	NOT EVALUATED	2/3	GOOD	46, XX [20]	0.5	Int-1
4	F	RA	<5%	ABSENT	0/1	GOOD	46, XX [20]	0	Low
5	F	RCMD	<5%	ABSENT	0/1	GOOD	46, XX [20]	0	Low
6	F	RCMD	<5%	ABSENT	2/3	GOOD	46, XX [20]	0.5	Int-1
7	F	RCMD	<5%	ABSENT	2/3	GOOD	46, XX [20]	0.5	Int-1
8	M	RAEB-2	5–10%	ABSENT	0/1	GOOD	46, XY [20]	0.5	Int-1
9	M	RA	<5%	ABSENT	0/1	GOOD	46, XY [20]	0	Low
10	M	RA	<5%	ABSENT	2/3	GOOD	46, XY [20]	0.5	Int-1
11	M	MDS-U	<5%	NOT EVALUATED	0/1	GOOD	46, XY [20]	0	Low
12	F	RA	<5%	NOT EVALUATED	0/1	GOOD	46, XX [20]	0	Low
13	F	RCMD	<5%	ABSENT	0/1	GOOD	46, XX [20]	0	Low
14	M	RA	<5%	ABSENT	0/1	GOOD	46, XY [20]	0	Low
15	M	MDS-U	<5%	MF-1	0/1	GOOD	46, XY [20]	0	Low
16	M	MDS-U	<5%	MF-1	0/1	GOOD	46, XY [20]	0	Low
17	M	RAEB-1	5–10%	ABSENT	0/1	INTERMEDIATE	47, XY, +8 [20]		Int-1
18	M	RA	<5%	NOT EVALUATED	2/3	GOOD	46, XY [20]	0.5	Int-1
19	M	RA	<5%	NOT EVALUATED	0/1	GOOD	46, XY [20]		Low
20	M	RCMD	<5%	ABSENT	2/3	GOOD	46, XY [20]	0.5	Int-1
21	F	RA	<5%	NOT EVALUATED	0/1	INTERMEDIATE	46, XX [20]	0.5	Int-1
22	M	RCMD-RS	<5%	NOT EVALUATED	2/3	GOOD	46, XY [20]	0.5	Int-1
23	F	RA	<5%	NOT EVALUATED	0/1	GOOD	46, XX [20]	0	Low
24	F	RCMD	<5%	ABSENT	0/1	GOOD	46, XX [20]	0	Int-1
25	F	RCMD	<5%	NOT EVALUATED	0/1	GOOD	46, XX [20]	0	Low
26	M	RA	5–10%	NOT EVALUATED	2/3	GOOD	46, XY [20]	1	Int-1
27	M	RA	<5%	ABSENT	2/3	GOOD	46, XY [20]	0.5	Int-1
28	M	RCMD	<5%	ABSENT	0/1	INTERMEDIATE	47, XY, +8 [20]	0.5	Int-1
29	F	RCMD-RS	<5%	NOT EVALUATED	0/1	GOOD	46, XX [20]	0	Low
30	M	RCMD	<5%	MF-1	0/1	INTERMEDIATE	46, XY [20]	0.5	Int-1
31	F	RA	<5%	NOT EVALUATED	0/1	GOOD	46, XX [20]	0	Low
32	M	RA	<5%	NOT EVALUATED	0/1	INTERMEDIATE	46, XY [20]	1	Int-1
33	F	RCMD	<5%	NOT EVALUATED	0/1	GOOD	46, XX [20]	1	Low
34	F	MDS-U	<5%	ABSENT	0/1	GOOD	46, XX [20]	0	Low
35	F	RA	<5%	NOT EVALUATED	0/1	GOOD	46, XX [20]	1	Low
36	M	RCMD	<5%	ABSENT	0/1	GOOD	46, XY [20]	0	Low

RCMD: Refractory Cytopenia with Multilineage Dysplasia. RA: Refractory Anemia. RAEB-1: Refractory Anemia with Excess of Blasts type 1. RAEB-2: Refractory Anemia with Excess of Blasts type 2. MDS-U: Myelodysplastic Syndrome—Undefined. RS: Ringed Syderoblasts.

**Table 2:** Relative risk according to clinical parameters and response to ESA.

Age	RR	0.65
	CI lower	0.28
	CI Upper	1.53
ECOG	RR	0.36
	CI lower	0.06
	CI Upper	2.17
Number of Cytopenias	RR	0.57
	CI lower	0.13
	CI Upper	2.54
% Blasts	RR	0.47
	CI lower	0.039
	CI Upper	5.7
BM Fibrosis	RR	0.38
	CI lower	0.029
	CI Upper	5.2
IPSS	RR	0.5
	CI lower	1.13
	CI Upper	1.92
Histology	RR	1
	CI lower	0.262
	CI Upper	3.82

**Table 3:** Adverse events are probably related to the study drug.

Adverse Event	Grading				
	I	II	III	IV	V
Infection			1 (3%)	1 (3%)	
Metabolic		1 (3%)			
Vascular		1 (3%)	3 (9%)		
Liver	1 (3%)				
Constitutional symptoms	1 (3%)				

## Data Availability

The data presented in this study are available on request from the corresponding author. The data are not publicly available due to privacy and ethical restrictions.
